# Two distinct β-sheet structures in Italian-mutant amyloid-beta fibrils: a potential link to different clinical phenotypes

**DOI:** 10.1007/s00018-015-1983-2

**Published:** 2015-07-21

**Authors:** Ellen Hubin, Stéphanie Deroo, Gabriele Kaminksi Schierle, Clemens Kaminski, Louise Serpell, Vinod Subramaniam, Nico van Nuland, Kerensa Broersen, Vincent Raussens, Rabia Sarroukh

**Affiliations:** 1 Nanobiophysics Group, Faculty of Science and Technology, MIRA Institute for Biomedical Technology and Technical Medicine, University of Twente, 7500 AE Enschede, The Netherlands; 2 Structural Biology Brussels, Department of Biotechnology (DBIT), Vrije Universiteit Brussel (VUB), Pleinlaan 2, 1050 Brussels, Belgium; 3 Structural Biology Research Center, VIB, Pleinlaan 2, 1050 Brussels, Belgium; 4 Laboratory of Structure and Function of Biological Membrane, Faculté des Sciences, Center for Structural Biology and Bioinformatics, Université Libre de Bruxelles (ULB), Campus de la Plaine CP 206/02, Boulevard du Triomphe, 1050 Brussels, Belgium; 5 Department of Chemical Engineering and Biotechnology, University of Cambridge, New Museums Site, Pembroke Street, Cambridge, CB2 3RA UK; 6 School of Life Sciences, University of Sussex, Falmer, East Sussex, BN1 9QG UK; 7 FOM Institute AMOLF, Science Park 104, 1098 XG Amsterdam, The Netherlands

**Keywords:** Amyloid-beta peptide, E22K mutation, Secondary structure, Fibril polymorphism, Tropism, β-sheet conformation

## Abstract

Most Alzheimer’s disease (AD) cases are late-onset and characterized by the aggregation and deposition of the amyloid-beta (Aβ) peptide in extracellular plaques in the brain. However, a few rare and hereditary Aβ mutations, such as the Italian Glu_22_-to-Lys (E22K) mutation, guarantee the development of early-onset familial AD. This type of AD is associated with a younger age at disease onset, increased β-amyloid accumulation, and Aβ deposition in cerebral blood vessel walls, giving rise to cerebral amyloid angiopathy (CAA). It remains largely unknown how the Italian mutation results in the clinical phenotype that is characteristic of CAA. We therefore investigated how this single point mutation may affect the aggregation of Aβ_1–42_ in vitro and structurally characterized the resulting fibrils using a biophysical approach. This paper reports that wild-type and Italian-mutant Aβ both form fibrils characterized by the cross-β architecture, but with distinct β-sheet organizations, resulting in differences in thioflavin T fluorescence and solvent accessibility. E22K Aβ_1–42_ oligomers and fibrils both display an antiparallel β-sheet structure, in comparison with the parallel β-sheet structure of wild-type fibrils, characteristic of most amyloid fibrils described in the literature. Moreover, we demonstrate structural plasticity for Italian-mutant Aβ fibrils in a pH-dependent manner, in terms of their underlying β-sheet arrangement. These findings are of interest in the ongoing debate that (1) antiparallel β-sheet structure might represent a signature for toxicity, which could explain the higher toxicity reported for the Italian mutant, and that (2) fibril polymorphism might underlie differences in disease pathology and clinical manifestation.

**Electronic supplementary material:**

The online version of this article (doi:10.1007/s00018-015-1983-2) contains supplementary material, which is available to authorized users.

## Introduction

The conversion of native and functional peptides or proteins into higher ordered, toxic aggregates, and eventually to amyloid fibrils, is characteristic of many human proteinopathies [1]. Amyloid fibrils deposit extra- or intracellularly and are implicated in neurodegenerative disorders and systemic amyloidoses [2]. The defining molecular unit of these amyloid fibrils is the cross-β spine that originates from extended β-sheets composed of β-strands that are arranged perpendicular to the fiber axis [3].

Although the cross-β characteristic is a common structural feature, amyloid fibrils show a great structural variety and can differ in their underlying structure, symmetry, width, twist periodicity, and curvature [4–6]. This structural polymorphism can have several molecular origins. First, fibril polymorphs can differ in the number of protofilaments (the minimal fibrillar entities) [7]. Second, distinct orientations and modes of association of protofilaments and patterns of inter-residue interactions determine how protofilaments are oriented [8–11]. Third, variations in the underlying protofilament substructure can contribute to fibril polymorphism [12, 13]. Despite the highly conserved arrangement of fibrils in a cross-β manner along the elongation axis, fibrils can thus display considerable heterogeneity and structural polymorphism.

The biological relevance of fibril polymorphism is not yet fully understood, but it is notable that fibril polymorphism has been reported for several disease-related proteins. Substantial evidence indicates that different fibril morphologies exert different toxicities in vitro and could be related to differences in disease pathology and progression in vivo, or could underlie the preference of amyloid to deposit in specific cellular locations (i.e., tropism) [13–23]. However, the link between fibril polymorphism and clinical subtypes of amyloidoses is still lacking.

One of the disease-related proteins for which fibril polymorphism has been reported is the amyloid-beta (Aβ) peptide [24]. The Aβ peptide is one of the underlying causes of Alzheimer’s disease (AD) [25, 26], inclusion body myositis [27], dementia with Lewy bodies [28], and cerebral amyloid angiopathy (CAA) [29–31]. Whereas Aβ in AD is primarily deposited in the brain parenchyma, and CAA is related to cerebrovascular amyloid deposition. Aβ deposition in vessel walls makes them prone to rupture and narrows their lumina to the point of occlusion, resulting in secondary lesions associated with CAA, such as intracerebral and subarachnoid bleeding, multiple infarcts, and periventricular edema [32]. CAA is, however pathophysiologically related to AD and found with high prevalence in AD patients (80–90 %) [33].

CAA is a common clinical symptom of early-onset familial AD (FAD), in which disease symptoms occur earlier in life compared to the more prevalent sporadic, late-onset AD [34]. Most mutations that are recognized to cause FAD are concentrated in the amyloid precursor protein (APP) either within or around the Aβ domain. Mutations clustering near the Aβ N-terminus were shown to alter Aβ production and enhance the kinetic of fibril and intermediate aggregate species formation [35, 36], while mutations located at the Aβ C-terminus were only shown to affect the release of Aβ by favoring acceleration of the production [37–40]. Interestingly, mutations reported within residues 21–23 of Aβ are implicated in increasing Aβ production, enhancing Aβ aggregation kinetic and/or delaying Aβ clearance [41–46].

Carriers of the Italian E22K [47], Iowa D23N [42], or Dutch E22Q [48] Aβ mutation predominantly display the clinical phenotype characteristic of CAA. In contrast, the Flemish A21G mutation results in both significant amyloid accumulation in brain blood vessels and parenchymal amyloid plaques [44], whereas Arctic E22G carriers develop progressive dementia, typical of AD, but without the severe CAA that characterizes other mutations in this region [49].

In this study, we monitored the aggregation of wild-type (WT) and Italian-mutant E22K Aβ_1–42_ under different experimental conditions, and structurally characterized the resulting fibrils. We provide evidence that, under near-physiological conditions, E22K Aβ_1–42_ spontaneously forms fibrils comprising stable antiparallel β-sheets, in contrast to WT fibrils that are composed of parallel β-sheets, similar to most amyloid fibrils described in the literature [50]. Moreover, to the best of our knowledge, this is the first experimental demonstration that the underlying β-sheet arrangement of Italian-mutant Aβ fibrils is altered upon a change in pH, and that inter-conversion of the corresponding fibril polymorphs occurs.

These results are interesting in light of the emerging view that (1) antiparallel β-sheet structure may be of importance in the pathology of AD [5, 51], and that (2) fibril polymorphism may be implicated in in vivo differences in terms of disease pathology and age of disease onset. We suggest that the antiparallel β-sheet conformation of E22K Aβ fibrils, and maybe other CAA-related Aβ peptides, predisposes them to mainly deposit in blood vessel walls, resulting in CAA.

## Materials and methods

### Reagents and chemicals

WT Aβ_1–42_ was purchased from American Peptide Co. (Sunnyvale, CA, USA), and E22K and D23N Aβ_1–42_ were purchased from JPT (JPT Peptide Technologies, Germany). Dimethyl sulfoxide (DMSO 99.9 % purity), hexafluoropropan-2-ol (HFIP), Thioflavin T (ThT), uranyl acetate, and pepsin were obtained from Sigma-Aldrich (St. Louis, MO, USA). Antibodies (6E10, 4G8, 12F4) were from Covance (Emeryville, CA, USA). Horseradish peroxidase-conjugated anti-mouse antibodies were purchased from Millipore (Billerica, MA, USA). Supersignal West Pico Chemiluminescent Substrate and ECL plus Western blot detection system were obtained from Thermo Fisher Scientific (Biotechnology, Rockford, IL, USA) and GE Healthcare (Piscataway, NJ, USA), respectively.

### Aβ sample preparation

Aβ peptides were dissolved in cold HFIP at a concentration of 2 mg/mL and incubated at room temperature (25 °C) for 1 h. HFIP was evaporated under nitrogen flow and residual HFIP was removed under vacuum using a Speed Vac (Thermo Savant). Prior to incubation, peptides were dissolved in DMSO at a final concentration of 5 mM and then diluted to a final concentration of 100 μM in TBS (Tris-buffered saline: 20 mM Tris/HCl, pH 7.4, 100 mM NaCl) or in 10 mM HCl pH 2.0. Peptides were incubated at 37 °C under quiescent conditions. Fibrillar samples were centrifuged for 30 min at 16,100*g* prior to analysis by Fourier transform infrared (FTIR) spectroscopy and hydrogen/deuterium exchange mass spectrometry (HDX-MS).

### Transmission electron microscopy

Aβ samples (5 µL of a 100 µM concentration) were adsorbed to carbon-coated Formvar 400-mesh copper grids (Agar Scientific) for 1 min. The grids were washed and stained with 1 % (w/v) uranyl acetate. Samples were studied with a JEOL JEM-1400 microscope (JEOL Ltd., Tokyo, Japan) at 80 kV. Transmission electron microscopy (TEM) images are representative of three independently prepared Aβ solutions.

### Atomic force microscopy

Atomic force microscopy (AFM) images were acquired using a VEECO Dimension 3100 atomic force microscope (Bruker), operated in tapping mode in air using silicon cantilevers with a resonance frequency of 325 kHz, a spring constant of 46 Nm^−1^, and a tip radius of 10 nm (µMASCH, NSC15/no Al). Images were collected at a scan rate of 1 Hz. Each fibrillar sample (5 µL of a 100 µM concentration) was deposited for 15 min onto freshly cleaved mica surfaces to enable adsorption. The samples were rinsed with ultrapure water (5 × 200 µL) and left to dry in air before imaging.

### Dot blot analysis with Aβ-region specific antibodies

Aβ aggregation was monitored by spotting 1 µg of Aβ onto a nitrocellulose membrane at several incubation times. The membranes were blocked for 1 h at 4 °C in 5 % non-fat dry milk in TBS-Tween 20 buffer and then incubated (24 h, 4 °C) with mouse monoclonal Aβ-region specific antibodies 6E10, 4G8, or 12F4 (all diluted 1:3000 in 0.5 % non-fat dry milk in TBS-Tween 20 buffer). Horseradish peroxidase-conjugated anti-mouse antibody (1:2000 in 0.5 % non-fat dry milk in TBS-Tween 20 buffer, 4 °C, 1 h) was used as secondary antibody. Detection was carried out using the Supersignal West Pico Chemiluminescent Substrate and the ECL^®^ Western blot kit. Images were recorded and analyzed using the ImageQuant 400 gel imager and ImageQuant TL software (GE Healthcare).

### Secondary structure and HDX measurements using ATR-FTIR spectroscopy

Attenuated total reflectance FTIR (ATR-FTIR) spectra were recorded on an Equinox 55 IR spectrophotometer (Bruker Optics, Ettlingen, Germany). A quantity of 2 µg of Aβ was spread on the diamond surface (2 × 2 mm) of the internal reflection element and was washed with excess milli-Q water to eliminate salts. Excess water was evaporated under nitrogen flow. Each spectrum represents the mean of 128 repetitions, recorded at a resolution of 2 cm^−1^. The ATR-FTIR data were analyzed using Kinetics software (SFMB, Brussels, Belgium) and processed for baseline correction and subtraction of the water vapor contribution. Spectra were smoothed at a final resolution of 4 cm^−1^ by apodization of their Fourier transform by a Gaussian line and intensities were normalized to the intensity of the major β-structure peak around 1630 cm^−1^. All depicted spectra were deconvolved using a Lorentzian deconvolution factor with a full width at half height (FWHH) of 20 cm^−1^, a Gaussian apodization factor with FWHH of 16.67 cm^−1^ to obtain a resolution enhancement factor of 1.2. Deconvolution increases the resolution of the spectra in the amide I region which is most sensitive to the secondary structure of proteins. Next, curve fitting was performed on the non-deconvolved ATR-FTIR spectra to determine the secondary structure content of each sample. The proportion of a particular structure is computed to be the sum of the area of all the fitted bands (having their maximum in the frequency region where that structure occurs) divided by the area of all the Lorentzian bands (having their maximum between 1700 and 1600 cm^−1^). They were chosen by *Kinetics* software based on the shape of the most deconvolved spectrum (α-helices and random coil: 1637–1662 cm^−1^, turn: 1662–1682 cm^−1^, β-sheet: 1613–1637 cm^−1^, and 1682–1689 cm^−1^). Curve fitting has a tendency to overestimate the β-sheet content. Combining curve fitting analysis and hydrogen/deuterium exchange on Aβ fibrils (7 days) enhances the prediction of secondary structure and allow us to determine exchange dynamics. The decay of the NH-associated amide II band (1520–1580 cm^−1^) was used to monitor the exchange of the amide group. Results were analyzed as previously described [52]. This information is of macroscopic nature and is related to the compactness of the amyloid fold.

### Thioflavin T fluorescence

Fibril formation was probed using ThT fluorescence as described previously [53]. Briefly, 5 µM of ThT was freshly dissolved in 50 mM glycine/NaOH pH 8.5 and 4.5 µg of Aβ was added to 1 mL ThT solution. ThT fluorescence emission was recorded between 460 and 560 nm (excitation wavelength of 450 nm). All measurements were recorded on a LS55 fluorimeter (PerkinElmer Instruments) at 25 °C with slit widths of 5 nm.

### X-ray fiber diffraction

Aβ fibrils (8 mg/mL) were aligned between wax-tipped capillaries and allowed to air dry [54]. The partially aligned fiber was placed on a goniometer head and data were collected using a Rigaku rotating anode source (CuKα) and Saturn 944+ CCD detector. The diffraction patterns were examined and measured using Clearer [55].

### HDX-MS coupled to pepsin proteolysis

A volume of 25 μL of amyloid fibrils of WT and E22K Aβ_1–42_, grown for 21 days in TBS (100 μM monomeric concentration), were collected by centrifugation at 16,100*g* for 30 min at 4 °C. A volume of 24 μL of supernatant was removed and the pellet was resuspended with 24 μL D_2_O. Labeling was carried out for 15 min at room temperature. Fibril samples were recovered by centrifugation at 16,100*g* for 30 min at 4 °C. Since then, samples and solutions were held on ice. A volume of 24 μL of D_2_O supernatant was removed and fibrils were dissolved in 20.4 μL of 100:0.5 (v/v) H_2_O/HCOOH containing pepsin (1:5, enzyme/substrate, w/w). After 20 s, 3.6 μL of 100:0.5 (v/v) MeCN/HCOOH was added [85:15:0.5 (v/v/v) H_2_O/MeCN/HCOOH final concentration] allowing fibril dissolution [56].

For HDX on monomers of WT and E22K Aβ_1–42_, the dried peptides were first dissolved in DMSO at a concentration of 2 mM and subsequently resuspended in D_2_O (final concentration: 80 μM) and exchange was allowed for 45 min. One μL of the exchanged monomeric peptide was added to 20.4 μL of 100:0.5 (v/v) H_2_O/HCOOH containing pepsin (1:5, enzyme/substrate, w/w). After 20 s, 3.6 μL of 100:0.5 (v/v) MeCN/HCOOH was added [85:15:0.5 (v/v/v) H_2_O/MeCN/HCOOH final concentration].

HDX was then evaluated by electrospray ionization mass spectrometry (ESI–MS). The deuterium content of the samples was analyzed by ESI–MS on a Q-TOF Ultima API spectrometer (Waters/Micromass). Samples were electrosprayed from gold-coated glass capillaries (Thermo Fisher). Capillary and cone voltages applied were 1.8 kV and 50 V, respectively. The same dead time (1 min) after mixing in protic solvent was used for all experiments. All measurements were performed in triplicate and mass spectra presented are averages of 20 s acquisition.

The fraction of D_2_O in the pepsin solution, used for monomeric and fibrillar samples, is 4 % (v/v) (1 μL D_2_O for 24 μL H_2_O). The labile terminal and side chain hydrogens exchange very rapidly even at pH 2.0–3.0, so that the final measured deuterium content should include an equilibrium distribution of deuterium into these sites. Because there are 27 and 28 such hydrogens in WT and E22K Aβ_1–42_, respectively, a total of only one deuterium atom (4 % of 27.5) should be incorporated at these sites for full-length monomeric Aβ and is thus not considered here. The deuterium content of the peptides is determined from the centroid of the molecular ion isotope peaks as described earlier [57]. The measured deuterium content in each peptide is corrected for back exchange (BE). Back exchange occurs during the pepsin processing of samples in solvent that contains exchangeable hydrogens, and during ionization itself, as previously described [58].

$$D_{\text{corr}} = m - {\text{MW}} + {\text{BE}}$$

$${\text{BE }} = {\text{MW }} + N{\,-\,}m_{100 \% }$$

*D*_corr_ is the corrected average number of amide deuterons after incubation in D_2_O, *m* is the measured centroid mass after 45 min of labeling, MW is the measured average molecular weight in H_2_O, *N* is the total number of amide hydrogens in each peptide (exchangeable sites), and *m*_100 %_ is the measured centroid peptide mass from 100 % deuteration controls. Monomeric Aβ is expected to be fully deuterated after only 30 min and is used as the control to estimate the back exchange during the analysis. The back exchange ranges from 12.5 to 37.8 % and is similar to other studies [10, 59].

## Results

### E22K Aβ_1–42_ forms fibrils with an antiparallel β-sheet conformation

**Fig. 1:**
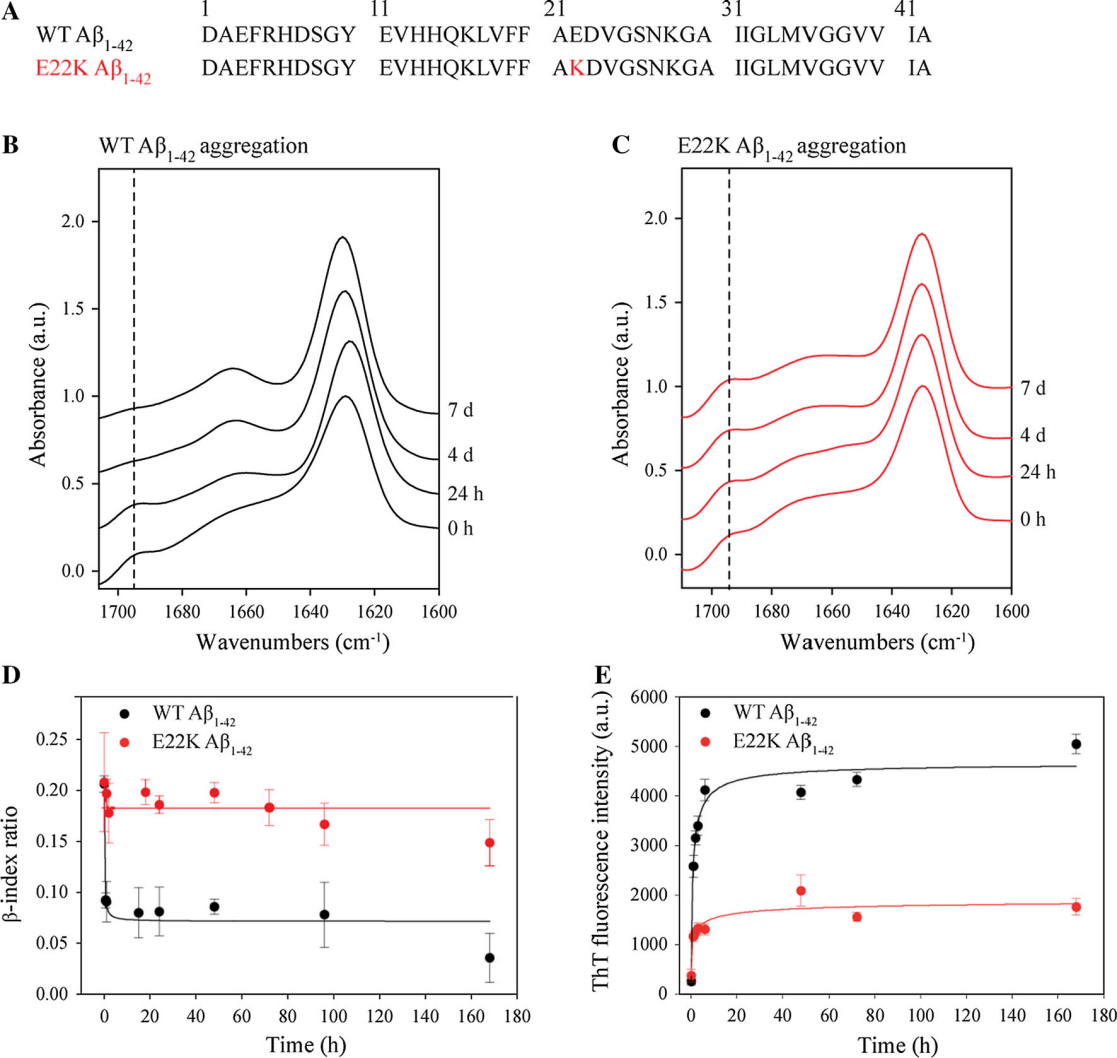
The Italian Aβ mutation induces formation of antiparallel β-sheet fibrils. **a** WT and Italian-mutant Aβ sequences. The replacement of a Glu residue for a Lys at position 22 (E22K) in the Italian mutant is indicated in *red*. **b**–**e** Aggregation of WT and E22K Aβ_1–42_ peptide in TBS pH 7.4 at 37 °C was monitored for 7 days using **b**–**d** ATR-FTIR and **e** ThT fluorescence. The amide I region (1700–1600 cm^−1^) of the ATR-FTIR spectra of **b** WT and **c** E22K Aβ_1–42_ are depicted and *vertical broken lines* are shown at 1695 cm^−1^. Spectral intensities were normalized to the intensity of the major contribution of β-structure around 1630 cm^−1^. Spectra were vertically offset for better visualization. Spectra were deconvolved using a Lorentzian deconvolution factor with a FWHH of 20 cm^−1^ and a Gaussian apodization factor with a FWHH of 16.67 cm^−1^ to obtain a resolution enhancement factor *K* = 1.2. Spectra are representative of at least three independent experiments. **d** The β-index ratio (1695/1630 cm^−1^ intensity ratio) was calculated on the basis of scaled ATR-FTIR spectra. Means and error bars have been calculated on the basis of three independent experiments. **e** ThT fluorescence emission at 485 nm (*λ*_ex_ = 450 nm) was measured during aggregation. Intensity was corrected for the ThT background. Means and error bars have been calculated on the basis of three independent experiments

WT and E22K Aβ_1–42_, of which the primary sequences are displayed in Fig. 1a, were dissolved in 100 % HFIP to ensure removal of pre-formed aggregates. After evaporation of HFIP, the aggregation of Aβ was induced by dissolving the peptide film in DMSO at a concentration of 5 mM. Peptide samples were then diluted to a final concentration of 100 µM in TBS buffer of pH 7.4. The emergence of heterogeneous oligomeric and prefibrillar species, and subsequent fibril formation for both WT and E22K Aβ_1–42_ was confirmed by TEM (Fig. S1A). AFM, being more sensitive for the detection of smaller Aβ aggregates (i.e., oligomers) than TEM [60], demonstrated the fibril preparations to be virtually oligomer free (Fig. S1B). Moreover, the accessibility of the C-terminus of both peptides was lost upon aggregation as assessed by dot blotting with region specific anti-Aβ antibodies (Fig. S2). This is in full agreement with previous nuclear magnetic resonance (NMR) studies showing folding of the C-terminus of the Aβ_1–42_ peptide into the fibril core [17, 61].

To gain insight into the structural rearrangements occurring during aggregation of the E22K Aβ_1–42_ peptide and into the differences with WT Aβ_1–42_, we monitored the Aβ aggregation process using ATR-FTIR (Fig. 1b, c). The amide I absorption band (1600–1700 cm^−1^) was used for IR spectra analysis because this band results from C=O stretching vibrations of peptide bonds and is most sensitive to changes in the H-bonding and secondary structure in proteins [52, 62]. Upon aggregation, WT and E22K Aβ_1–42_ both displayed β-sheet structure, indicated by a major peak around 1630 cm^−1^ (Fig. 1b, c). The position and width of this peak have been related to the number of β-strands and/or the formation of H-bonds [63–65]. Both peptides also showed an additional minor peak around 1695 cm^−1^ at early aggregation time points (0, 24 h). This peak, in conjunction with the major peak, is the signature of an antiparallel arrangement of β-strands [66–68] and can be attributed to the presence of oligomers [69–72]. Mature fibril formation was marked by a high β-sheet content for both WT and E22K Aβ_1–42_, as demonstrated by curve fitting of the amide I region (Table 1). However, the structural rearrangements occurring during the oligomer-to-fibril transformation were different for WT and E22K Aβ_1–42_. As demonstrated previously, the oligomer-to-fibril transformation of WT Aβ_1–42_ was accompanied by a major change in secondary structure [71]. The antiparallel contribution associated with the minor peak around 1695 cm^−1^ disappeared when fibrils were formed (Fig. 1b), indicating reorganization of β-strands from an antiparallel to a parallel orientation [71]. In contrast, in the case of E22K Aβ_1–42_, the 1695 cm^−1^ peak persisted in time, suggesting that Italian-mutant fibrils are mainly composed of antiparallel β-sheets (Fig. 1c). The structural differences occurring during aggregation were quantified by the β-index ratio (defined as the 1695/1630 cm^−1^ intensity ratio), which has been shown to be proportional to the percentage of antiparallel arrangement of β-strands in a β-sheet [66, 69, 71, 72]. The β-index ratio of WT Aβ_1–42_ decreased significantly during the oligomer-to-fibril transformation (0.20 ± 0.01 to 0.03 ± 0.02 after 7 days), whereas the E22K β-index ratio retained high values throughout aggregation (0.21 ± 0.05 to 0.15 ± 0.03 after 7 days) (Fig. 1d). High β-index values have been reported to be characteristic for antiparallel β-structured proteins such as avidin, concanavalin A, and bacterial outer membrane porin F [69, 70]. These results suggest that, contrary to most structures reported previously for amyloid fibrils, Italian-mutant Aβ_1–42_ fibrils display an antiparallel β-sheet architecture.

**Table 1:** Distinct HDX and secondary structure contributions for WT and E22K Aβ_1–42_ fibrils

ATR-FTIR				HDX-MS coupled to pepsin proteolysis			
Secondary structure (%)		Exchanged amide protons (%)	β-index ratio	Pepsin-induced Aβ fragments	Exchanged amide protons	Protected NH/total	Protected NH/total (%)
WT Aβ_1-42_ fibrils							
β-sheet	63	30 ± 5	0.07	[1–19]	14.0 ± 0.5	4/18	22
				[20–42]	7.3 ± 0.7	14.7/22	67
α-helix	11						
				[35–42]	3.1 ± 0.5	3.9/7	56
Random coil	7						
Turn	19						
E22K Aβ_1-42_ fibrils							
β-sheet	58	50 ± 5	0.19	[1–19]	14.3 ± 0.4	3.7/18	20
				[20–42]	9.6 ± 0.8	12.4/22	56
α-helix	5						
				[35–42]	3.4 ± 0.3	3.6/7	51
Random coil	11						
Turn	26						

HDX was determined using ATR-FTIR (during a time lapse of 1 h) and ESI–MS (during a time lapse of 45 min) coupled to pepsin proteolysis. Secondary structure contributions were estimated using ATR-FTIR

**Fig. 2:**
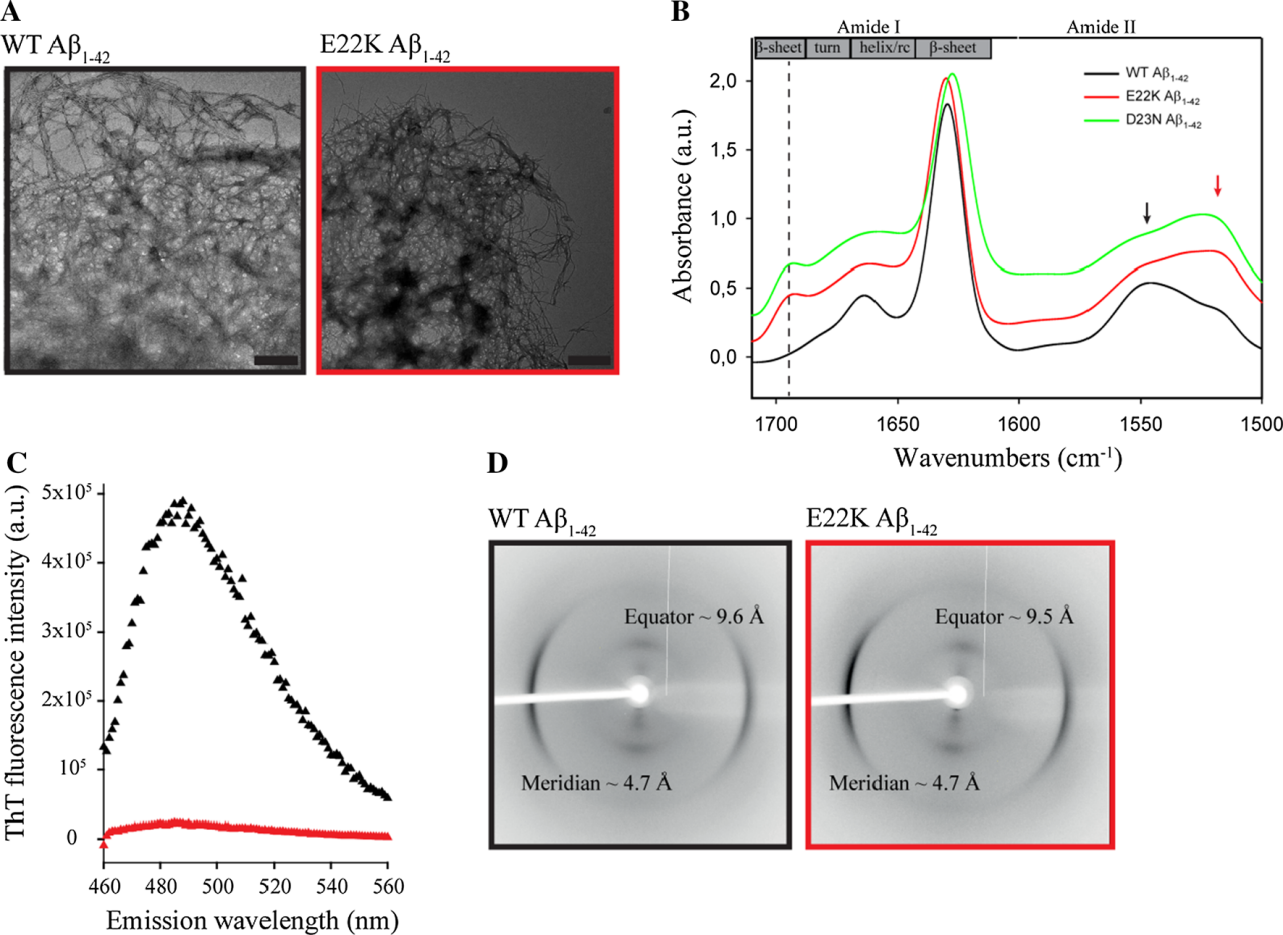
WT and E22K Aβ_1–42_ fibrils display similar morphologies but distinct underlying structures. WT and E22K Aβ_1–42_ fibrils (depicted in *black* and *red*, respectively) were structurally characterized after 7 days of incubation in TBS pH 7.4 at 37 °C. **a** TEM revealed dense networks of negatively stained fibrils for both fibril types. *Scale bars* represent 500 nm. **b** ATR-FTIR spectra of E22K, D23N, and WT Aβ_1–42_ fibrils, harvested after 30 min of centrifugation at 16,100*g*. E22K and D23N fibrils displayed an additional peak around 1695 cm^−1^ (*vertical dashed line*) and a shift in the amide II band to lower wavenumbers (as depicted by the *arrows*), indicative of an underlying antiparallel β-sheet architecture, compared to the parallel orientation of β-sheets in WT fibrils. Spectral intensities were normalized to the intensity of the major contribution of β-structure around 1630 cm^−1^. Spectra were vertically offset for better visualization. Spectra were deconvolved using a Lorentzian deconvolution factor with a FWHH of 20 cm^−1^ and a Gaussian apodization factor with a FWHH of 16.67 cm^−1^ to obtain a resolution enhancement factor *K* = 1.2. **c** ThT fluorescence emission spectra (*λ*_ex_ = 450 nm) corresponding to fibrils in **b**. E22K Aβ_1–42_ fibrils showed low reactivity with the fluorescent probe ThT, whereas WT fibrils induced a high ThT fluorescence signal. Spectra were corrected for the ThT background. **d** X-ray fiber diffraction resulted in the cross-β reflections, characteristic of amyloid fibrils [54] for both fibril types

To structurally characterize E22K and WT Aβ_1–42_ fibrils in more depth and to ensure samples were oligomer-free, fibrillar samples (7 days) were centrifuged at 16,100*g* for 30 min and the fibril pellet was resuspended in water prior to IR data collection. Dense amyloid networks with protruding, negatively stained fibrils were visualized using TEM for both fibril pellets (Fig. 2a). In contrast to WT Aβ_1–42_, the IR spectrum of the E22K Aβ_1–42_ fibril pellet still displayed the minor peak at 1695 cm^−1^ and a shift in the amide II band region (1500–1600 cm^−1^) to lower wavenumbers (Fig. 2b), features both corresponding to antiparallel β-sheet structure [71]. As oligomers were removed by the centrifugation procedure, this observation provides evidence that the antiparallel structural signature detected in the total aggregated E22K Aβ_1–42_ sample after 7 days of incubation (Fig. 1c) is due to E22K Aβ_1–42_ fibrils, as these are the main aggregation species at this time point. To validate this attribution, we recorded an IR spectrum for the fibril pellet of Iowa D23N Aβ_1–42_, shown previously to form metastable fibrils composed of antiparallel β-sheets [73], which presents identical IR spectral features (Fig. 2b).

The distinct underlying conformations of WT and E22K Aβ_1–42_ fibrils were further marked by different sensitivities for staining with ThT, a dye commonly used to detect amyloid fibrils [53, 74]. Whereas WT Aβ_1–42_ fibrils displayed a high ThT fluorescence intensity, the ThT fluorescence of E22K Aβ_1–42_ fibrils was significantly lower (Figs. 1e, 2c). Lower levels of ThT fluorescence could be indicative of a lower affinity of Italian-mutant fibrils for ThT, as shown previously for the Japanese ΔE22-Aβ_1–39_ mutant, and/or of less accessibility of the dye to potential binding sites [75].

As neither ATR-FTIR nor ThT fluorescence spectroscopy provided information on the relative positioning in the three-dimensional space, we resorted to X-ray diffraction. The latter revealed that both fibril types displayed meridional (4.7 Å) and equatorial (9.6–9.7 Å) reflections, corresponding to the distance between β-strands within one β-sheet, and to the distance between β-sheets, respectively (Fig. 2d). These reflections are consistent with the cross-β diffraction pattern that is characteristic for amyloid fibrils [3]. However, the orientation obtained was not sufficient to distinguish between parallel and antiparallel fibril structures. Furthermore, it is difficult to establish antiparallel β-sheet signatures from X-ray fiber diffraction due to a systematic absence of even numbered repeats arising from the 2_1_ helix [76].

ATR-FTIR and fluorescence spectroscopy data indicate that WT and E22K Aβ_1–42_ fibrils contain different β-sheet organizations. Further analysis was then performed to gain more detailed understanding of their structural differences.

### The central region is more exposed in E22K than in WT Aβ_1–42_ fibrils

HDX patterns of fibrils were measured using ATR-FTIR spectroscopy to gain insight into the H-bonded β-sheet network. Hydrogen atoms exchange most easily when not involved in H-bonds and/or when they are not buried in the fibril core. Estimates of the secondary structure contributions, based on the analysis of IR spectra (Fig. 2b), indicated that E22K Aβ_1–42_ fibrils contain slightly less β-sheet and more intrinsic disorder and turn contributions compared to WT fibrils (Table 1). Accordingly, E22K Aβ_1–42_ fibrils demonstrated a higher HDX ratio than WT fibrils: 50 (±5) % of amide hydrogens were exchanged by deuterium compared to 30 (±5) % for WT fibrils, during a time lapse of 1 h (Table 1). The difference in total HDX between WT and mutant fibrils corresponds to the backbone of eight amino acids, but the specific region responsible for this difference cannot be derived from this dataset.

**Fig. 3:**
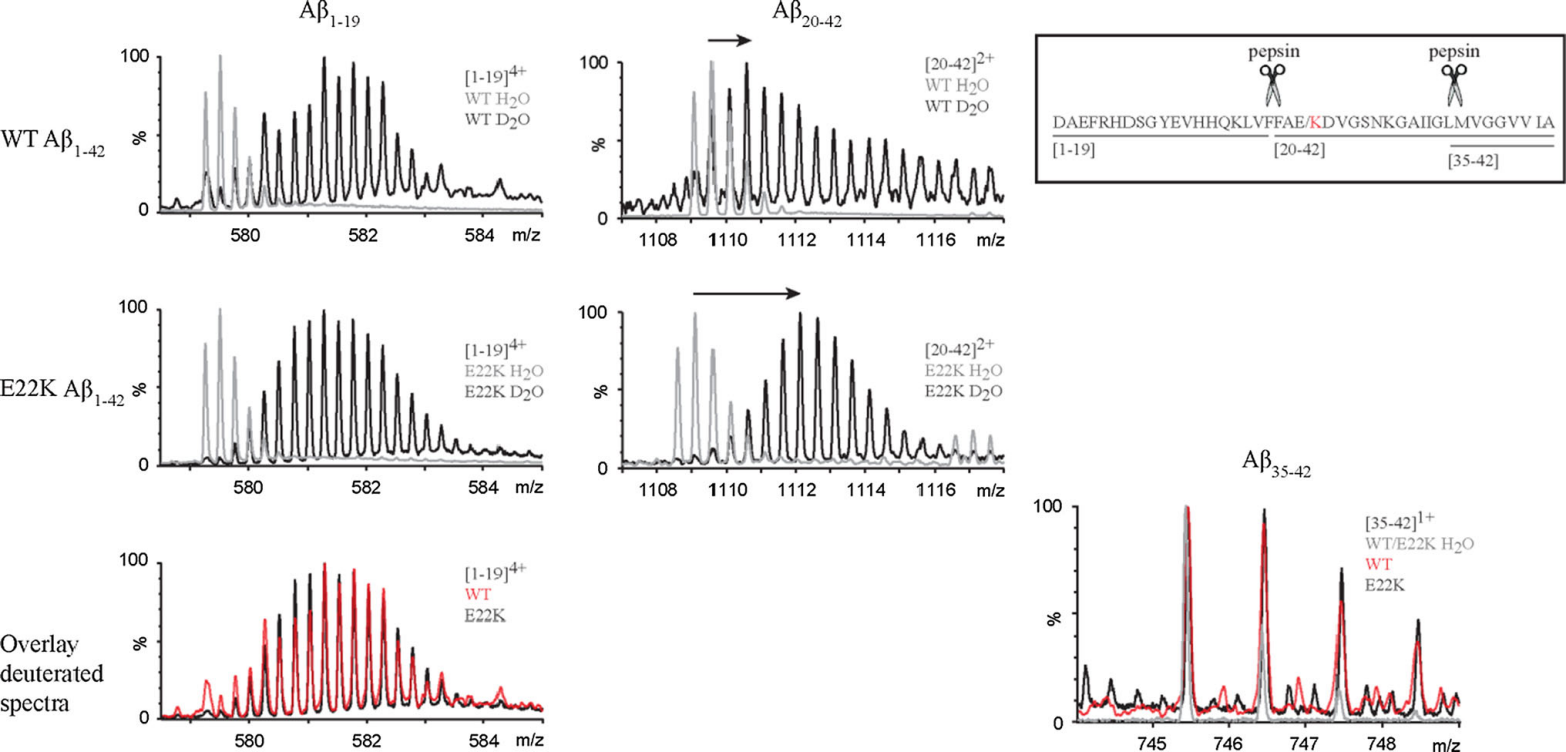
The central region is more exposed in E22K than in WT Aβ_1–42_ fibrils. Segmental HDX information of E22K and WT Aβ_1–42_ fibrils was revealed by HDX-MS coupled with pepsin proteolysis. Mass spectra corresponding to E22K and WT Aβ_1–42_ fibrils are displayed before (*gray*) and after (*black*) deuteration for 45 min. The charge state of each fragment is indicated in each panel. The mass spectra of the N-terminal fragment [1–19] and C-terminal fragment [35–42] were very similar after deuteration for both WT and E22K indicating no difference in deuterium incorporation in these regions. Conversely the mass spectra for the fragment [20–42] showed more exchange for E22K compared to WT Aβ_1–42_, as seen by the more pronounced mass increase after deuteration (indicated by *arrows*)

To achieve more insight into regional structural differences, additional segmental exchange information of fibrils was therefore revealed by HDX-MS coupled with pepsin proteolysis (Fig. 3). We identified three proteolytic Aβ fragments comprising residues [1–42], covering the whole sequence of the peptide. The mass spectra of the N-terminal fragment [1–19] and C-terminal fragment [35–42] were similar for both WT and E22K fibrils after HDX, indicating no difference in deuterium incorporation in these regions within the time frame of the exchange. In contrast, the mass spectra for the fragment [20–42] showed that E22K Aβ_1–42_ fibrils were more exchanged than WT fibrils in this region as the increase in mass after deuteration was more pronounced. The number of deuterium atoms incorporated in each Aβ fragment was measured and data were corrected for back exchange. The corrected numbers of backbone amide protons exchanged after 45 min incubation in D_2_O are summarized in Table 1. For both WT and E22K Aβ_1–42_, the N-terminal region [1–19] showed little protection (respectively 22 and 20 %) in comparison to the C-terminal region (respectively 67 and 56 %) which is in agreement with the dot blotting results (Fig. S2) and data obtained by others using NMR for WT Aβ_1–42_ [17, 61]. The deuterium incorporation in regions [1–19, 35–42] showed no significant difference, as qualitatively observed from the mass spectra (Fig. 3). For peptide fragment [20–42], the solvent accessibility data resulted in (9.6 ± 0.8) total exchanged amide protons for E22K, which is significantly higher than the value obtained for the WT peptide (7.3 ± 0.7). Assuming that there is no difference in labeling in peptide [35–42], the difference in deuterium incorporation between WT and E22K Aβ_1–42_ fibrils can thus be localized to the central region [20–34] of the peptide.

### A change in pH reveals different β-sheet conformations for E22K Aβ_1–42_ fibrils

The antiparallel β-structured fibrils that have been recently reported for the Iowa mutant D23N Aβ_1–40_ peptide were suggested to be thermodynamically metastable [73]. In contrast, Italian-mutant fibrils displayed a high β-index ratio for several months that reached approximately 60 % of its original value after 1 year of incubation under near-physiological conditions (TBS pH 7.4), with the majority of the fibrils still retaining the antiparallel β-sheet conformation (Fig. S3).

**Fig. 4:**
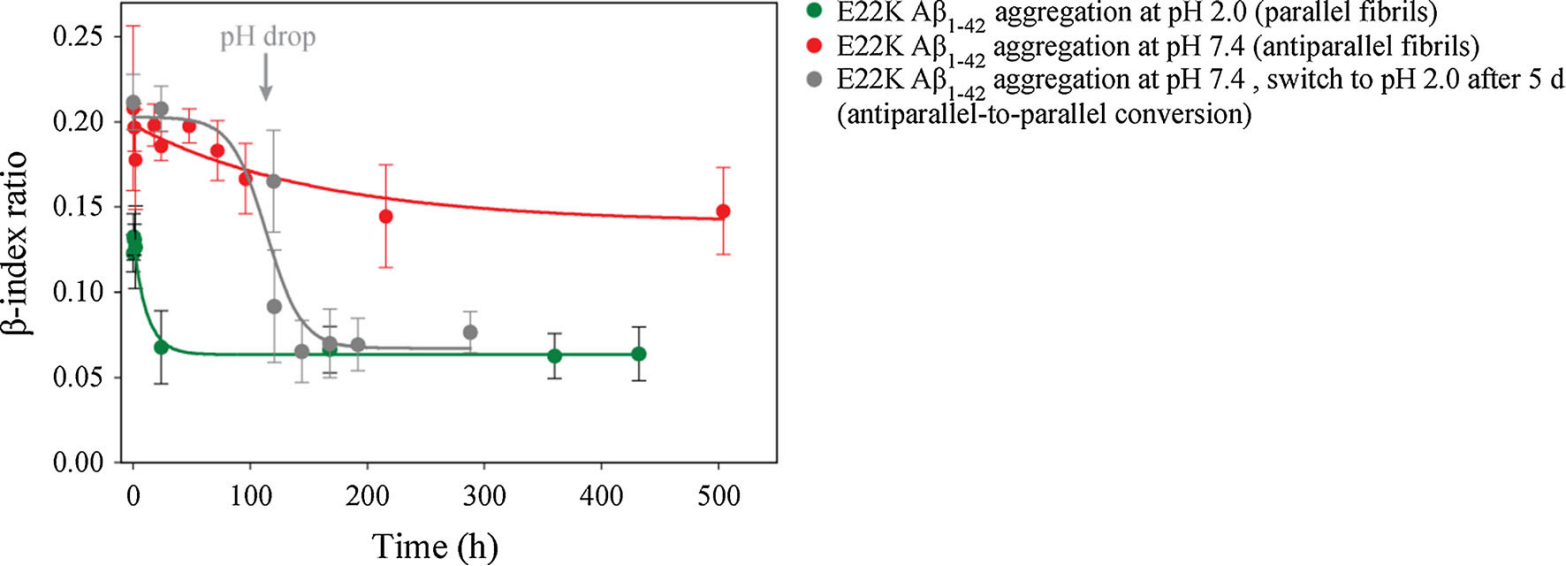
The β-sheet structure of Italian-mutant Aβ_1–42_ fibrils is pH sensitive. β-index ratios of E22K Aβ_1–42_ obtained during aggregation in TBS pH 7.4 (*red* *curve*) and 10 mM HCl pH 2.0 (*green curve*) at 37 °C show that antiparallel β-sheet fibrils are formed at neutral pH while parallel β-sheet fibrils are formed in acidic conditions. Moreover, fibrils grown at neutral pH undergo a structural change upon a shift to acidic pH (antiparallel-to-parallel transition) as seen by the significant decrease in β-index ratio (*gray curve*)

To gain more insight into the interactions that play a role in antiparallel β-sheet formation, we studied the behavior of E22K Aβ_1–42_ under different experimental conditions. Aggregation of E22K Aβ_1–42_ was monitored at low pH (10 mM HCl pH 2.0) at 37 °C. It has been suggested that WT Aβ fibrils are stabilized by a salt bridge between D23 and K28 [13, 17], whereas a salt bridge between K22 and D23 would be present in E22K Aβ fibrils [77]. These salt bridges would however not occur at low pH due to neutralization of the negative charge of the D23 side chain (pH < pKa of Glu/Asp), and this might elicit structural changes during the aggregation process. Accordingly, ATR-FTIR analysis revealed a WT-like behavior for the aggregation of the Italian mutant directly incubated at pH 2.0. The β-index ratio decreased significantly from 0.12 ± 0.01 to 0.06 ± 0.02 during the time course of the experiment (18 days), indicating a conversion from antiparallel oligomers to parallel fibrils (Fig. 4). Hence, the E22K Aβ peptide evolves into the parallel β-sheet fibrillar state at low pH, possibly due to alterations of electrostatic interactions.

Next, E22K antiparallel fibrils were first grown at neutral pH (TBS pH 7.4) and 37 °C, and the fibril pellet (obtained after centrifugation for 30 min at 16,100*g*) was then redissolved in 10 mM HCl pH 2.0. This pH jump lead to a significant decrease in the β-index ratio from 0.21 ± 0.01 to 0.08 ± 0.01, corresponding to parallel β-structured fibrils, indicating that an antiparallel-to-parallel conversion occurred and demonstrating that the inter-conversion of fibril polymorphs is possible. This decrease of the β-index ratio occurred within the dead time of the experiment (~30 min).

## Discussion

Amyloid oligomers have emerged as the culprit species responsible for potent toxicity activities [78, 79]. However, despite the amount of data published supporting their role(s) in neuronal degeneration and cell death, the molecular mechanism of toxicity is still puzzling [80]. Even so, oligomers exist in equilibrium with fibrils and therefore the kinetic, thermodynamic, and physiological factors affecting the formation, metabolism, and activity of one of these assemblies also affect the others. Aβ fibrils are inherently stable but are not inert and neither, non-toxic end product deposits. Although the toxicity of oligomeric species is much more pronounced [81, 82], Aβ fibrils do have a significant established effect on neuronal viability compared to unaggregated peptide [60]. By themselves, fibrils present direct detrimental effects in particular contexts (e.g., obstructive vascular amyloid, mechanical effect on membrane cells) [83–85] and can induce a neuroinflammatory response triggered by the activation of microglial cells [86] which is currently thought to contribute to AD progress [87].

In this manuscript, we report that the E22K mutation of Aβ oligomers shows a similar antiparallel β-sheet structural arrangement which is retained upon fibril conversion. This observation raises the question if indeed an antiparallel β-sheet can be regarded as a structural fingerprint for toxicity. Interestingly, in the particular case of FAD-related Aβ_1–42_ mutants, in vitro studies have demonstrated that neurotoxicity correlates well with the ability of these peptides to aggregate [88, 89].

### Antiparallel β-sheet structure: a key organization in amyloid aggregates?

The impact of antiparallel β-sheet structure on amyloids is still under debate, but evidence emerges that this structural signature can be attributed to several higher toxicity intermediates or off-pathway aggregates of the amyloid formation pathway. Streltsov and co-workers [90] reported the first crystal structure of oligomers of the p3 fragment, the N-terminally truncated Aβ variant, and provided evidence for their antiparallel arrangement. Later, the research team of Eisenberg revealed the structure of an off-pathway, cylindrical oligomer of a segment of the amyloid-forming protein αB-crystallin that resembled a β-barrel composed of six antiparallel β-strands [51]. We and other groups demonstrated that amyloidogenic proteins, such as Aβ, pass through an antiparallel β-sheet structured state, corresponding to oligomers, before undergoing a transition in structure to parallel β-sheet fibrils [69–71, 91–93]. Recently, the group of Tycko demonstrated that Aβ_1–40_ containing the Iowa D23N mutation, resulting in a predominant vascular phenotype, can assemble into antiparallel β-sheet structured fibrils that are thermodynamically metastable and have a ribbon-like appearance [73]. As these aggregates were all described to be transient and toxic, the antiparallel β-sheet arrangement has been suggested to represent a unique toxic signature [72].

In this study, we report that an antiparallel β-sheet signature is shared by oligomers and fibrils comprising the Italian-mutant E22K Aβ_1–42_ peptide (Figs. 1, 2). The antiparallel β-sheet fibrils are formed spontaneously under near-physiological conditions and display a high β-index ratio for at least 1 year (Fig. S3). The Italian-mutant Aβ peptide can thus potentially provide a major source of neurotoxicity, either in the form of soluble and diffusible oligomers that are considered the main toxic agents in AD [79, 94, 95], or in its fibrillar form as a trigger of neuroinflammation [96]. One may speculate that, together with the imbalance between the production and clearance of the mutated peptide, this unique structural signature may be linked to the early-onset and aggressive progression of the associated FAD. Accordingly, the Italian Aβ mutant shows increased pathogenicity compared to the WT Aβ peptide and is up to tenfold more toxic to cerebrovascular smooth muscle cells [97] and PC12 cells in vitro [88, 89], supporting its role in CAA.

### The Italian Aβ mutant can form antiparallel and parallel β-sheet fibrils

Amyloid fibrils share the cross-β spine motif, but their quaternary arrangement can differ due to distinct non-polar interactions; i.e., Van der Waals forces and aromatic packing, and polar interactions, i.e., electrostatic and H-bonding interactions. It is conceivable that substitution of a glutamic acid to a lysine at position 22 within the Aβ sequence may profoundly affect the pattern of interactions that determines its fibrillar structure. The residue at position 22 is part of the central Aβ region that poses the critical limiting step leading to the formation of the β-hairpin, the ordered β-turn-β structural organization characteristic of Aβ monomers within the fibril [89, 98, 99]. Based on solid state NMR data, Masuda and co-workers [77, 100] suggested previously that intermolecular β-sheet contacts in E22K Aβ_1–42_ fibrils are key events driven by this turn region.

The results obtained at pH 7.4 give more insight into the possible effects on the fibril structure due to the charge alteration in this central Aβ region. First, we demonstrate that the E22K mutation results in differences in IR spectra that reflect distinct H-bonding organizations of WT and E22K Aβ_1–42_ fibrils, i.e., parallel and antiparallel β-sheet arrangements respectively. The differences in structure of WT and E22K Aβ_1–42_ fibrils were further marked by different ThT fluorescence intensities and HDX behaviors (Figs. 1, 2, 3). Second, the E22K mutation most likely induces changes in the pattern of stabilizing electrostatic interactions, as it can interfere with salt bridges that have been suggested to occur within the monomeric unit of the fibrillar structure, e.g., between D23 and K28 [13, 17], but potentially also between K16 and E22 [13], and E22 and K28 [101]. Differences in electrostatic interactions could underlie the structural alterations demonstrated for E22K fibrils at different pH values (Fig. 4). Third, the E22K mutation may potentially influence conserved hydrophobic contacts within the monomer unit composing the fibril, e.g., between F19 and G38 [102], or affect the interdigitation of β-sheets that is responsible for the steric zipper interface underlying the cross β structure [1]. The steric hindrance induced by the large side chain of K22 may result in changes in the exposure of side chains to the outer fibril surface and contribute to packing polymorphism. Accordingly, the E22K mutation induced changes in solvent accessibility around the central region, as assessed by HDX measurements (Table 1; Fig. 3).

Furthermore, we provide evidence that structural alterations occur for E22K Aβ fibrils in different environmental growing conditions. At neutral pH, the antiparallel β-sheet fibrillar structure is favored. In contrast, the E22K Aβ_1–42_ peptide forms fibrils with a parallel β-sheet arrangement at low pH (Fig. 4). To the best of our knowledge, this is the first experimental demonstration that a mutated form of the full-length Aβ peptide can form amyloid fibrils with two different β-sheet structures in a pH-dependent manner. This structural diversity might be due to neutralization of charges of amino acid side chains involved in key fibril contacts, as discussed in the previous section. Moreover, our results show inter-conversion of antiparallel-to-parallel β-sheet Italian-mutant fibrils when changing from neutral to acidic pH conditions. Recently, Tycko and co-workers [103] demonstrated the evolution of a mixture of two Aβ fibril polymorphs to the thermodynamically most stable polymorph. It remains to be discovered whether the inter-conversion of Italian-mutant fibril polymorphs demonstrated in this work is due to internal structural rearrangement, or whether the antiparallel β-sheet fibrils are destabilized upon a decrease in pH and are rebuilt in a parallel β-sheet arrangement.

A detailed molecular understanding of the intermolecular interactions that dictate the quaternary structure of Italian-mutant Aβ is still lacking, but the results presented here suggest that differences in the H-bonding pattern, electrostatic interactions, and balance of multiple side chain contacts may underlie fibril polymorphism for this Aβ mutant.

### Pathophysiological relevance of different β-sheet architectures

It has been suggested that Aβ fibril polymorphism may have biological significance. Fibril polymorphism could underlie in vitro differences in neurotoxicity, or in vivo differences in disease pathology and progression in different individuals/cell types and/or types of amyloidosis (i.e., tropism) [13–23, 104]. Recently, the first detailed look at the architecture of amyloid fibrils from patient brains demonstrated that two AD patients with distinct clinical histories possessed Aβ fibrils with a different underlying structure [16]. Moreover, Prusiner and co-workers showed that mice inoculated with brain homogenates from an Arctic AD case exhibited a pathology that could be distinguished from mice inoculated with Swedish or sporadic AD samples. This was seen by differential accumulation of Aβ isoforms and distinct morphology of cerebrovascular Aβ deposition [105]. It now becomes clear that several mutations in the central Aβ region that are associated with CAA, including the Italian E22K and Iowa D23N mutations, can result in the formation of Aβ fibrils with an antiparallel β-sheet structure (Fig. 2b). This unique structural signature might predispose them to deposit in cerebral blood vessels, rather than mainly accumulating in plaques, as seen for WT Aβ. It remains to be solved how these in vitro observations of structural differences can translate into differences in vivo, but one possibility might be through distinct interactions with receptors responsible for Aβ clearance across the blood–brain barrier.

## Conclusions

In conclusion, the Italian-mutant Aβ peptide forms oligomers and fibrils in vitro that share the antiparallel β-sheet organization. Our results are particularly interesting in light of the ongoing debate that suggests that the antiparallel β-sheet signature might provide the potential detrimental toxic effect of Aβ. Moreover, this is the first study that experimentally demonstrates structural plasticity for E22K Aβ fibrils. The structural differences of WT and E22K Aβ fibrils observed in vitro might be a direct implication for their in vivo differences: WT and E22K Aβ deposition in extracellular plaques and cerebral blood vessel walls, respectively, and therefore associated with late- and early-onset AD, respectively.

### Electronic supplementary material

Below is the link to the electronic supplementary material.

Supplementary material 1 (DOCX 2488 kb)

## Abbreviations

AD: Alzheimer’s disease
AFM: Atomic force microscopy
ATR: Attenuated total reflectance
Aβ: Amyloid-beta peptide
CAA: Cerebral amyloid angiopathy
DMSO: Dimethyl sulfoxide
ESI: Electrospray ionization
FAD: Familial Alzheimer’s disease
FTIR: Fourier transform infrared
HDX: Hydrogen/deuterium exchange
HFIP: Hexafluoroisopropanol
MS: Mass spectrometry
NMR: Nuclear magnetic resonance
TBS: Tris-buffered saline
TEM: Transmission electron microscopy
ThT: Thioflavin T
WT: Wild type
