# Supplementary material for: Two distinct β-sheet structures in Italian-mutant amyloid-beta fibrils: a potential link to different clinical phenotypes
Source: Cell Mol Life Sci. 2015 Jul 21;72(24):4899–913. doi: 10.1007/s00018-015-1983-2 (PMC4648968; doi:10.1007/s00018-015-1983-2)
Supplement: Supplementary file 1 — Supplementary material 1 (DOCX 2488 kb) [file 18_2015_1983_MOESM1_ESM.docx]

**Supplementary information**

**Two distinct β-sheet structures in Italian mutant amyloid-beta fibrils:**

**a potential link to different clinical phenotypes**

Ellen Hubin^1,2,3^, Stéphanie Deroo^4^, Gabriele Kaminksi Schierle ^5^, Clemens Kaminski^5^, Louise Serpell^6^, Vinod Subramaniam^1,7^, Nico van Nuland^2,3^, Kerensa Broersen^1^, Vincent Raussens^4^, Rabia Sarroukh^4^

**Affiliations:**^1^ Nanobiophysics Group, MIRA Institute for Biomedical Technology and Technical Medicine, Faculty of Science and Technology, University of Twente, 7500 AE Enschede, The Netherlands
^2^ Structural Biology Brussels, Department of Biotechnology (DBIT), Vrije Universiteit Brussel (VUB), Pleinlaan 2, B-1050 Brussels, Belgium
^3^ Structural Biology Research Center, VIB, Pleinlaan 2, B-1050 Brussels, Belgium
^4^ Center for Structural Biology and Bioinformatics, Laboratory of Structure and Function of Biological Membrane, Faculté des Sciences, Université Libre de Bruxelles (ULB), Campus de la Plaine CP 206/02, Boulevard du Triomphe, B-1050, Brussels, Belgium  ^5^ Department of Chemical Engineering and Biotechnology, University of Cambridge, New Museums Site, Pembroke Street, Cambridge CB2 3RA, United Kingdom
^6^ School of Life Sciences, University of Sussex, Falmer, East Sussex BN1 9QG, United Kingdom
^7^ FOM Institute AMOLF, Science Park 104, 1098 XG Amsterdam, The Netherlands

**Corresponding authors:**

Broersen K (Tel: +31534893655; Email: [k.broersen@utwente.nl](mailto:k.broersen@utwente.nl); Fax: +33534891105)
Raussens V (Tel: +3226505386; Email: [vrauss@ulb.ac.be](mailto:vrauss@ulb.ac.be); Fax: +3226505382)

**This supplementary information contains three figures.**

**
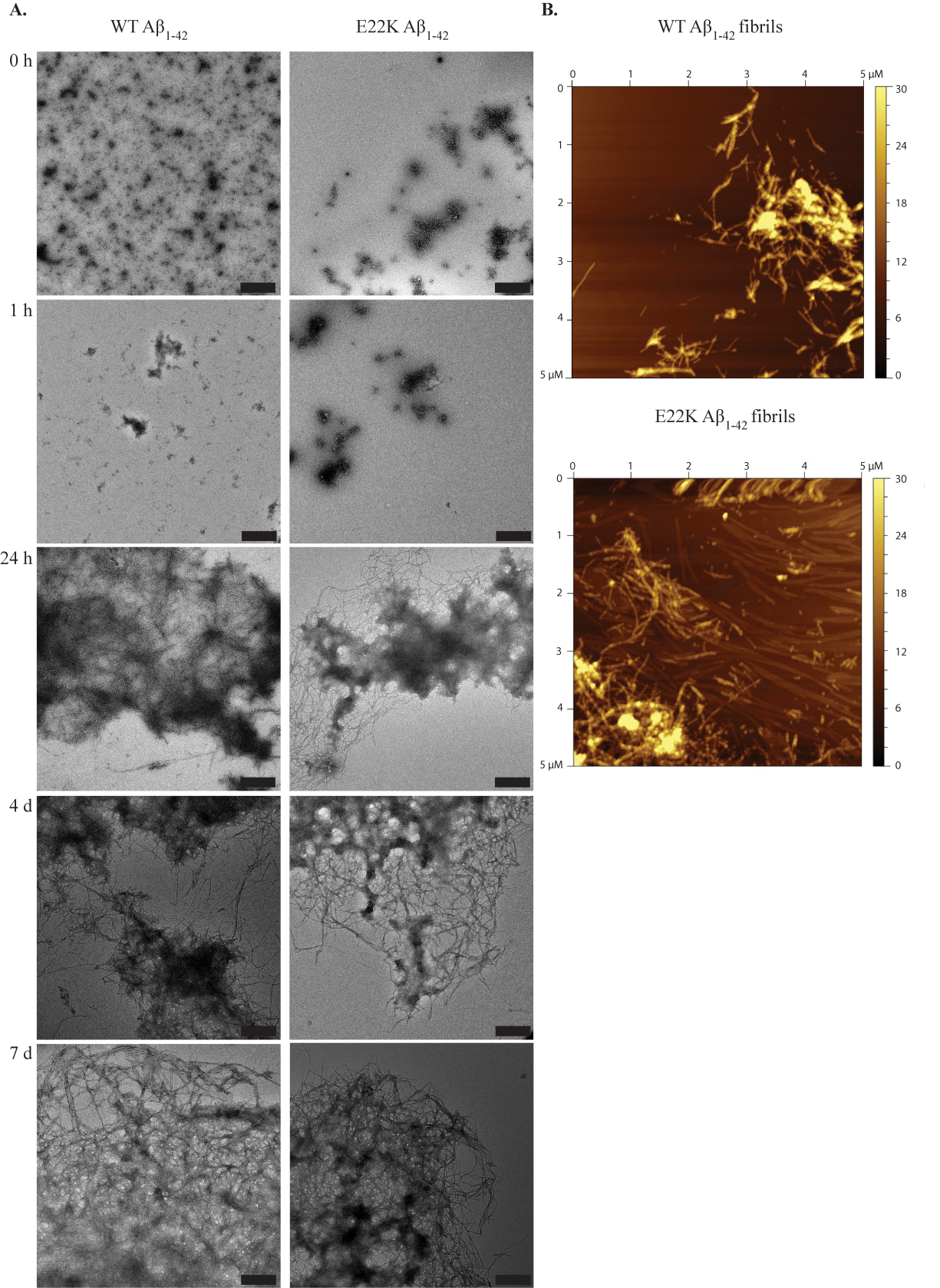
**

**Figure S1: WT and E22K Aβ_1-42_ fibrils display similar morphologies. (A)** The aggregation of WT and E22K Aβ_1-42_ at 37 °C was monitored using TEM. At early time points of Aβ aggregation, only spherical oligomeric and small thread-like prefibrillar species were detected for both peptides. WT and E22K Aβ_1-42_ then evolved into dense fibril networks. Scale bars represent 500 nm. **(B)** AFM characterization of both fibril types after 7 d of incubation at 37 °C.

**
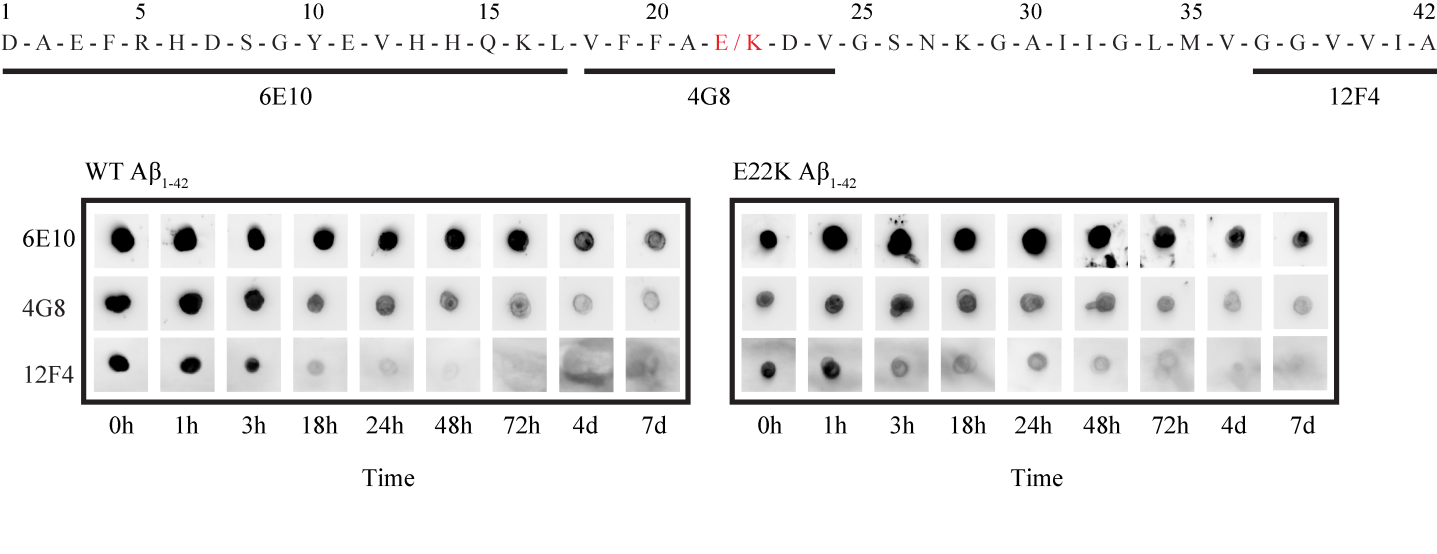
**

**Figure S2: The Aβ peptide C-terminus becomes inaccessible for antibody detection upon fibril formation.** Aggregation of WT and E22K Aβ_1-42_ at 37 °C was monitored using dot blotting with three Aβ region-specific monoclonal antibodies (6E10, 4G8, 12F4). Detection with region-specific antibodies was comparable for both peptides, with the C-terminus of the Aβ peptide becoming inaccessible upon fibril formation.

**
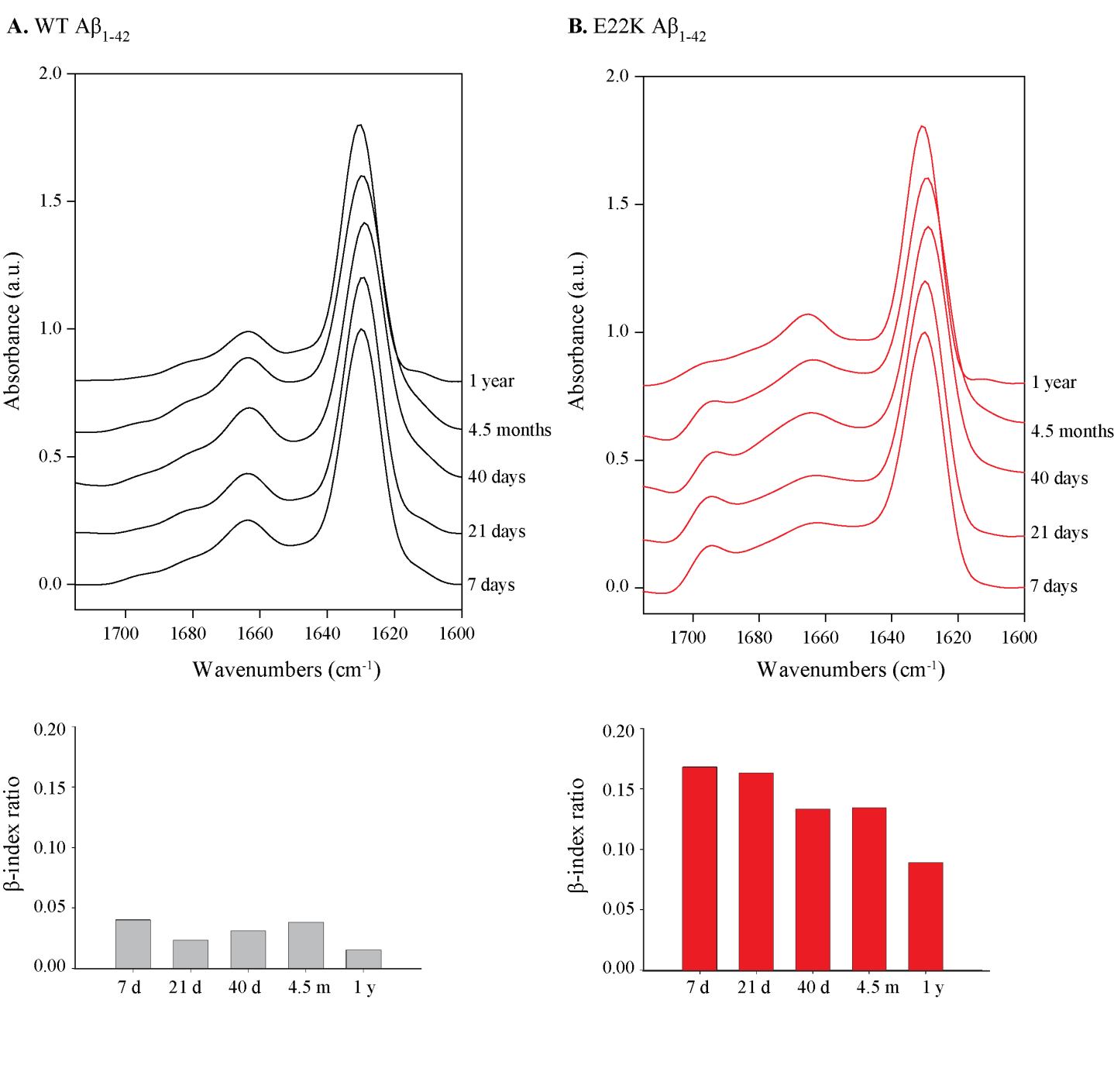
Figure S3: β-index evolution of WT and E22K Aβ_1-42_ fibrils.** The structural properties of **(A)** WT and **(B)** E22K Aβ_1-42_ fibrils were monitored using ATR-FTIR during 1 year. Spectral intensities were normalized to the intensity of the major contribution of β-structure around 1630 cm^-1^. Spectra were vertically offset for better visualization. Spectra were deconvolved using a Lorentzian deconvolution factor with a FWHH of 20 cm^-1^ and a Gaussian apodization factor with a FWHH of 16.67 cm^-1^ to obtain a resolution enhancement factor K = 1.2. **(Insets)** The β-index ratio (1695/1630 cm^-1^ intensity ratio) was calculated on the basis of scaled ATR-FTIR spectra.
